# Cooperative leadership as a condition for patient-reported rehabilitation success

**DOI:** 10.3389/fresc.2023.1114666

**Published:** 2023-03-15

**Authors:** Thorsten Meyer, Vera Kleineke, Maren Stamer

**Affiliations:** ^1^ Institute for Rehabilitation Medicine, Medical School, Martin-Luther-University Halle-Wittenberg Halle (Saale), Germany; ^2^ School of Public Health, Bielefeld University, Bielefeld, Germany; ^3^ German Pension Insurance North, Luebeck, Germany; ^4^ Alice Salomon Hochschule Berlin, University of Applied Sciences, Berlin, Germany

**Keywords:** rehabilitation, teamwork, collaboration, cooperation, success, qualitative study

## Abstract

**Introduction:**

Rehabilitation is a complex intervention that takes place in a complex setting. The MeeR project (characteristics of successful rehabilitation facilities) aims to identify complex conditions of successful rehabilitation outcomes.

**Methods:**

A project with a sequential mixed-methods study design with a quantitative prestudy and a qualitative main study was applied. In the quantitative study, quality assurance data of the German Pension Insurance was used to (1) develop and compute a multifacet z-standardized outcome index based on patient-reported outcome data, (2) rank *k* = 273 orthopedic rehabilitation facilities comprising *n* = 112,895 patients and *k* = 86 cardiac rehabilitation institutions comprising *n* = 30,299 patients based on their outcome index score by means of a league table, and (3) adjust the ranking by basic patient characteristics (age, gender, diagnosis, weeks out of work prior to rehabilitation, application for pension). In the qualitative main study, *k* = 6 rehabilitation facilities (orthopedic and cardiac rehabilitation centers) were recruited based on the results of the quantitative analysis: three facilities that ranked top 10% and three facilities that ranked lowest 10% of the adjusted league table. All six rehabilitation facilities were visited each for 1 week by two researchers. We conducted participant observations, expert interviews with medical and administrative leaders, group discussions with rehab team members, and group discussions with patients. Subsequently, a systematic comparison of the results of the upper and lower 10% facilities was conducted to identify those characteristics that distinguished those institutions from one another.

**Results:**

One of the three clusters of characteristics that distinguished the above and below 10% facilities related to teamwork or interdisciplinary cooperation: among others, the extent of interdisciplinary cooperation was higher in the rehabilitation facilities with a higher degree of success, the leading medical doctors were less dominant in these institutions, and there was also a more comprehensive representation of the team within team meetings, i.e., the quality and amount of interdisciplinary cooperation were higher in these institutions compared to rehabilitation facilities with a lower level of success.

**Discussion:**

This project provided qualitative evidence for the role of interdisciplinary cooperation and collaborative leadership and its different facets for patient-related successful rehabilitation in orthopedic and cardiac rehabilitation. It provides valuable insights into the fabric and structure of a rehabilitation institution and a variety of target points for team development and group-leading interventions.

## Introduction

In Germany, medical rehabilitation is mainly provided by intensive multidisciplinary complex services in rehabilitation facilities specialized in different patient groups of short-term duration (usually 3–4 weeks in the context of somatic medical rehabilitation) through mostly inpatient care with the option of after-care services in the community. The era of assessment and accountability (1) has strongly influenced the present rehabilitation system in Germany since a comprehensive quality assurance and management system was introduced in the early 1990s (2). There has been considerable effort in the documentation of structure and process quality, and a number of different studies have provided early evidence on care variability among rehabilitation facilities (3–9). Outcome quality has only played a marginal role in that time, although, as a well-known German rehabilitation researcher noted in 2007, in the end, the proof (of rehabilitation services) is in the pudding (i.e., in the rehabilitation successes) (10). Patients should be able to expect similar rehabilitation services, including success rates, irrespective of the rehabilitation facility they are admitted. Studies reported differences in rehabilitation outcomes in the areas of rehabilitation after hip or knee replacement (11), cardiac rehabilitation (12), psychosomatic rehabilitation (13), and chronic back pain (8, 14). However, outcome variation in terms of success seemed to be less pronounced than the difference in structural or process quality (5, 15).

We use the term *success* in a specific sense (16). It is different from *efficacy* or *effectiveness* in the sense that it does not need proof based on an experimental study design, which is the prerequisite of the latter terms. Patients can *experience* a program or intervention as successful. Also, those responsible for patient care can judge a program as successful when it is able to reach certain predetermined goals. We define *success* as the documented appraisal of single, relevant endpoints or an aggregate appraisal of different relevant endpoints that are attributed to clinical intervention, are interpreted as effects of this intervention, and are made from a specified perspective, e.g., from a patient’s or physician’s perspective. Indicators of success are found in quality assurance programs, observational studies, or applications of direct measurement of change, e.g., transition ratings (17).

Rehabilitation service facilities differ in the degree of success on the level of patients’ outcomes. These differences can only partly be accounted for by different patient characteristics, including clinical and functioning characteristics (e.g., different diagnostic groups, duration of work absence, and proportion of patients applying for disability pension). Therefore, the question of how to explain differences among rehabilitation service organizations with regard to patient-level success remains unanswered. In preparation for our study, we have conducted a narrative review on potential relevant characteristics of rehabilitation facilities for patient success [published in German within a report, see (18)]. The structural aspects of quality were the size of the organization (in terms of the number of beds, number of cases, and number of staff members), characteristics of staff members (e.g., qualifications, team composition, and motivation of team members), technical infrastructure, characteristics of premises/rooms, financial capacities, presence and type of quality management system, food, and spare-time offers. Also, different aspects of process quality were identified, such as ways of admission, diagnostics, care concepts, dealing with rehabilitation goals, characteristics of the process of care/therapies (e.g., continuation of care), or after-care. Also, aspects of organizational culture (e.g., mission and vision, philosophy, and working atmosphere), patient-centeredness and staff-centeredness, external and internal communication, and patient–professional interaction were identified in the review. The dimensions identified within the development of an International Classification of Service Organizations in Rehabilitation [ICSO-R (19, 20)] could serve as a framework for relevant quality aspects of rehabilitation institutions, too. However, we know of just one other study within the German rehabilitation healthcare system that systematically related organizational characteristics of the rehabilitation facility to patient outcomes in an empirical study (21). They found indications for a relationship between the network social capital of the rehabilitation team and patient outcome in terms of functional capacity at the end of rehabilitation based on a standardized approach. Consequently, we have developed a broad research framework for our study comprising structural characteristics, processes, communication and cooperation, and conceptual characteristics potentially related to patient-level rehabilitation success that was set up to sensitize the empirical part of our study.

The aim of the present paper is to report on the results of a qualitative project, which was set up to identify factors that account for differences between inpatient rehabilitation services on an organizational level related to rehabilitation success on a patient level. The report focuses on aspects of *cooperation and teamwork* in rehabilitation clinics, which is one of the three themes identified within the larger project (18).

## Methods

The main idea of the MeeR project^1^ was to select six rehabilitation facilities in total, three of them substantially above and three of them below average with regard to patient success, to conduct visitations in these facilities for the duration of a working week each, and to systematically compare the above- and below-average facilities to identify potential factors that could account for the differences among the rehabilitation facilities with regard to patient success. The complete study design is reported in (18). The study started with a written survey of rehabilitation staff members in *k* = 80 orthopedic (random sample) and *k* = 80 cardiac rehabilitation facilities. Based on the results of this survey, a workshop comprising *n* = 23 different stakeholders of the rehabilitation field, i.e., staff members of the German Pension Insurance, CEOs of rehabilitation facilities, and representatives from quality management and research, was held to set up the theoretical perspectives for the visitation phase of our project. These included sensitizing concepts like working staff orientation and satisfaction of staff members, organization of the facility (infrastructure, processes, quality management), interdisciplinary cooperation, patient orientation and satisfaction (with a special emphasis on aspects of participation), communication and motivation, especially enhancement of motivation, philosophy of the institution and the rehabilitation concept, aspects to be considered prior and after the rehabilitation phase, and the integration and cooperation of the institution with funding bodies and regional networks. Based on quality assurance data of the German Pension Insurance (2) (*N* = 30,441 cardiac patients and *N* = 113,284 orthopedic patients), we set up case-mix-adjusted league tables ranking all rehabilitation facilities using a composite outcome index. From these league tables, we identified those clinics that belonged to the *upper and lower 10%* of the league table. Of 11 rehabilitation facilities contacted, we reached the respective number of *k* = 6 rehabilitation facilities willing to participate in the study. Research team members were blind to the results of the allocation of the clinic (i.e., upper or lower level of patient success). (Members of the) Rehabilitation facilities were assured that their participation in the project would not be made public. They were offered a face-to-face presentation and discussion of study results relating to all six participating rehabilitation facilities.

The sampling procedure resulted in two orthopedic clinics and one cardiac rehabilitation clinic belonging to the upper 10% of the league table, and two orthopedic clinics and one cardiac rehabilitation clinic belonging to the lower 10% of the league table that were visited by two research team members for the duration of one working week. We applied a mix of qualitative research methods: a group discussion with patients, a group discussion with rehabilitation team members, an expert interview with the leading physician and the leading administrator, and participatory observations (22–25).

In the observations, we followed both a patient and rehabilitation team member perspective. After informed consent, we followed patients during their admission procedures and therapies. Staff was followed during therapies, team meetings, or daily routines. In addition, we took part in different additional occasions depending on the respective situation (e.g., cafeteria, waiting zone, and smoking zone). In principle, there was no video or audio taping during the observations. Observations were documented *post hoc* based on prespecified documentation sheets. These documentation sheets consisted of a common head to label the situation, characterize time (date, time, and duration of observation; and time of note taking) and place (including arrangements, if necessary), characterize people involved and their respective functions, and characterize possible specifics of the situation. This was followed by a place for open field notes related to different broad issues as defined by the sensitizing concepts (see above), e.g., communication and motivation, interdisciplinary cooperation, patient orientation and satisfaction, working staff orientation, and satisfaction of staff members. Also, a field for additional notes not fitting to the categories provided was added. Analysis of participatory observations was based on thematic analysis (24, 26).

All group discussions and interviews were audio-taped (when possible and agreement was present) and transcribed verbatim. Group discussions with patients were conducted using a moderation guide that aimed to elicit patient-related experiences regarding different aspects of patient handling and care. Questions were focused on experiences of the initial clinical investigation, goal setting, different therapies, communication and interaction with staff members, and contacts with fellow patients. We tried to evoke self-perpetuating discussions using open questions and—possible controversial—quotations taken from prior research as points of departure. Group discussions with rehabilitation team members were set up to include all medical and therapeutic professionals and nursing and social work professionals. While we asked not to include senior physicians in the group discussions, senior physicians actually took part in every (!) staff group discussion. Questions were focused on experiences with the patients and other professionals, especially focusing on issues of interprofessional teamwork. We also used quotations from prior research as points of departure to elicit discussions among the participants. Group discussions were set up to last 90 min. Analysis of group discussions was based on thematic analysis. Also, an in-depth analysis of group discussions with rehabilitation team members was used by applying the method of documentary analysis, which is especially suitable for analyzing interactions (22, 23, 27).

Individual expert interviews were conducted with the chief physician and the administration manager. Questions of the interview guide focused on aspects of patient orientation (e.g., openness toward individual needs and transfer to the everyday life of patients), philosophy of work (e.g., rehabilitation concept, quality management, and organization of work), staff orientation (qualifications and motivation), and communication and cooperation. The duration of interviews differed with regard to the time available, usually from 60 to 90 min. On one occasion, the researcher was invited to the room of the administration manager, but it turned out that he was not willing to conduct an interview, and we were not able to document the contents of our conversation. Analysis of interviews was primarily based on thematic analysis (24).

In principle, the analysis followed an explorative approach using broader concepts to guide our questions and observations as a point of departure and, at the same time, being open to new emerging themes or topics. Analysis of the different qualitative data was conducted in four steps. First, a comprehensive thematic analysis of the data was conducted to get an overview of the different topics appearing in the data (26). We then set up six comprehensive descriptions of the rehabilitation facilities based on the themes identified. Then, we used these descriptions to compare the facilities systematically and come up with similarities and differences. Within this phase, we moved beyond a simple content analysis approach and tried to identify bigger underlying themes in a reconstructive approach that helped to understand the differences we saw among the facilities (23). After unblinding the group status of the rehabilitation facilities, we elaborated on the differences between the rehabilitation facilities above and below average regarding patient-level success. The aim of this last phase was to develop empirically grounded hypotheses on the differences between successful and less successful rehabilitation clinics. The whole approach was stimulated by grounded theory methodology, especially the systematic comparison and the aim to develop empirically founded hypotheses (28).

Ethical approval was granted by the Ethics Committee of the Hannover Medical School.

## Results

### General result

Before we move to the results relating to interdisciplinary work, we have to point out that the differences between the clinics from the upper and lower parts of the league table were not as obvious, simple, or unequivocal as might have been expected. We could not identify a single characteristic that could distinguish the different institutions. In every rehabilitation facility, we were grateful to meet staff members highly engaged in the care of their patients on a personal level. We had to delve deeper into our analysis to make the complex and sometimes subtle differences visible. They relate to three domains labeled (1) interdisciplinary cooperation; (2) rehabilitation goal setting; and (3) design of rehabilitation services in respect of patient and staff members. Within this paper, we will focus on the first domain.

### Participation of team members in team meetings

The involvement of team members in team meetings is more pronounced in rehabilitation facilities with higher levels of success. Team meetings are appreciated to a higher degree in these facilities.

All organizations have in common that physicians represent the only professional group whose complete participation in team meetings goes without saying. Other professional groups might only be represented by means of delegates, often the leading person of the respective group. This is the case in two of the three institutions with a lower level of patient success but in none of those with an upper level of patient success. Team members who are not able to take part in the team sessions regularly criticize this type of delegation. For example, a physiotherapist from a facility with a lower level of patient success reports in a group discussion:

*And probably it would be good sometimes, it is, clearly, just not always possible here, I would say with eighteen therapists altogether in our team, that we can sit together within the team and take part in team meetings or case reports all together, I do favour it when those involved with the patient, when just now, when you know ahead of time in the Thursday meeting we will talk about this patient, that the respective therapist maybe could just take part in that meeting. Since it is always better to say something in person, compared to letting it done by our team leader*. (B2/GDST/FC/181–191)^2^

This goes along with the perceptions of the staff members that these team sessions are not appreciated enough. An occupational therapist from another facility with a lower level of patient success states in a group discussion:

*But I really miss those Monday meetings, where every therapist had taken part, I shall say.* (B1/GDST/FC/88–89)

Observational protocols of these team meetings indicate that team meetings applying a delegate model were not used effectively. Although team members from different professions come together, in these meetings, a mutually shared ground for interdisciplinary communication was hardly accomplished.

*The same female physician now presents a patient (case history). The male senior physician calls this patient an interesting case, it was about a scoliosis that has been corrected late in life. The x-rays are shown, at the same time everybody is talking to each other, not together within the group, but in one-to-one conversations.* (B1/OP/team meeting)

*The meeting starts with a salutation from the chief physician. Then the handover of the physician from the night shift takes place. He talks immensely fast and muted, a one-to-one conversation with the chief physician follows directly afterwards, in which no other person is involved. Even the eye-contact of both is restricted to their conversation.* (B1/OP/early team meeting)

However, these results only relate to two of the three rehabilitation facilities with a lower level of patient success. This limitation makes clear that differences in success cannot simply be reduced to a single obvious characteristic. Still, in rehabilitation facilities with an upper level of patient success, therapists and physicians are completely represented without using the respective leading managers. In one of these three clinics, they applied a partial delegate model. Here, not every team member took part in the team meeting, but the respective group sent different delegates (not only the leading manager) to these meetings. Within these institutions, participation in interdisciplinary team meetings is positively appraised and both valued by the staff members and the leading managers. A psychologist states in a group discussion:

*(…) I am really happy to be part of the team meetings or the early meetings, that take part every day, since from a psychological perspective it is also important to know, of course, how they* [i.e. the patients] *behave within the other therapies, in the occupational therapy, how do they present themselves in the teaching kitchen?* (A1/GDST/FE/144–149)

A leading administrator states in an interview:

*Yes, and this topic meeting or exchange is a very important topic in our clinic × *[name of the facility] *there is exchange on the different levels, but it is within a lot of meetings that take place, is always, well, interdisciplinary and not just, it does not just stick within the departments, but there are always many disciplines involved.* (A3/IVLA/245–251)

### Modality of interdisciplinary cooperation

The degree and mode of reciprocity in interdisciplinary cooperation are more pronounced in rehabilitation facilities with an upper level of patient success. In rehabilitation facilities with a lower level of patient success, we found that communication of the different team members is concentrated toward the physicians. Therapists and nurses forwarded information mainly to the physicians.

*The physiotherapist recommends removing a patient from the so-called shoulder-group. Also at this point, her mode of speech appears low, cautious and hesitant. She introduces the issue, if it would not be better to exclude all patients with a “bad” cervical spine status from the shoulder group. The leading physicians, however, stand for a different approach, i.e. they hold that all patients with cervical spine syndrome should take part in the shoulder group. (…) The issue is not further elaborated on or discussed, the physicians` statement determines the further conduct. No opposition can be observed.* (B3/OP/team meeting)

Orders came from the physicians directed to the staff members of the other health professions.

*Well, when first talk to each other and for me the final word always comes from the physician. That's how I forward it to the patients, too. Even when I do have a different opinion, and I talk with doctor P* [name of physician] *about it, then I just make my own thoughts, well, I see it differently, but to the patient I say that the doctor has said somehow or other, and we stick to it.* (B3/GDST/FG/874–879)

We found this hierarchical order also expressed in the seating arrangements in team meetings.

*The interdisciplinary team meeting takes part in the office of the chief physician. This office is about 25 m² big and comprises a treatment bed, a sink, a sideboard, a table with about eight chairs and a big writing desk. The physicians sit at the big round table, two places are still vacant. The physiotherapist, the occupational therapist, the dietician and the social worker sit in the background on the treatment bed, which makes most of the physicians at the table to turn their back on them.* (B1/OP/team meeting)

There were indications that this lack of reciprocity in interdisciplinary work emanated both from the physicians themselves and from the self-conception of the team members of the other health professions. The following example results from the opportunity to observe different staff members, situations, or fields of action within our study and to relate these situations to each other. It stands for lack of or a failed interprofessional interaction.

*A nurse realizes that a patient is still taking a medication for gastric protection despite the fact that he had discontinued to take his painkiller. She states that tomorrow will be the ward round of the leading physician, then this could be clarified. She does not make a note in the patient records. This, so she says, is not a nursing task. She assumes that this would transcend her competencies.* (B1/OP/nursing)

A lack of interprofessional communication that appears to be intrinsically related to a self-appreciation of one's professional role results in futile or even harmful care.

*We were present at the next morning in the ward round. Nobody noticed that the patient still takes a medication for gastric protection that appears not to be necessary any more. (B1/OP/ward round)*

In sum, we noticed a lesser amount or even lack of cooperation in rehabilitation facilities with lower levels of patient success.

On the contrary, we noticed more intense, reciprocal professional communication across the different professions in rehabilitation facilities with upper levels of patient success. For example, in these facilities, the team meetings were not merely dominated by physicians. Other professionals participated in or initiated discussions on patients without being called upon.

*The chief physician then asks the physiotherapists about their experiences with the patient from a physiotherapist`s perspective. A physiotherapist reports what has been done so far. She makes proposal how to proceed further, these proposals are being taken up by the physicians. The dietician then asks, without being addressed beforehand, about the nutritional state of the patient. A response is provided by the nurses. The review of the patient is concluded thereby.* (A2/OP/team meeting)

Therapists approach physicians with a proposal for further therapeutic actions. The following situation has been observed in a facility with upper levels of patient success within an early morning meeting of physicians.

*Somebody knocks at the door and a female physiotherapist enters. A seat is offered to her and she gets seated. Shortly after that, the leading physician hands over to her. She says she is here because of the stool group 2. She inquires if the physicians all are aware about the difference between stool group 1 and 2. Hereupon she explicates the meaning of this difference and asserts that there are frequent mismatches of patients in the groups. She assures herself that everybody has understood her delineation. She also asks if everybody knows how to manage the correct assignment of patients within the management system. Some nod, others do not react. The physiotherapist delivers a prepared handout to every physician that explains the correct assignment of patients into the groups. Hereupon she expresses her gratitude and leaves the room.* (A3/OP/early meeting of physicians)

Even patients of these facilities perceived that therapeutic decisions were not exclusively in the hands of the physicians. A patient from the same facility states during the group discussion:

*You do not notice some differences among physicians, nurses, therapists, I have not noticed them. I sense that, wherever I say something, if it is related to health they align themselves, every opinion counts apparently, team decisions et cetera.* (A3/GDPT/FE/1289–1293)

Still, even in these institutions, decisions that are important for interdisciplinary work are made by physicians, too. Staff members of different professions can contribute to these decisions in a substantial way. Decisions have to be transparent and justified in these institutions. We have observed that professional controversies were made transparent and discussed openly.

*(…) We do lead a lot of open discussions. Even when it is* ((he clears his throat)) *about strategies how to deal with patients, everybody can engage in the discussion. When I have the feeling that everybody has given a comment, then a final decision has to be taken. And this decision is taken based on best arguments that have been put forward by each individual. (…)* (A3/INLP/160–166)

### Amount of physicians` dominance in light of interdisciplinary cooperation

In every rehabilitation facility we visited, we found a dominant role of the physicians within the rehabilitation team when it came to the distribution and ascription of responsibilities. Still, within the rehabilitation facilities with a lower level of patient success, we could notice a more comprehensive and pronounced dominant role of the physicians. Both aspects we have elaborated on before, i.e., the organization of the team meetings and the related reciprocity of interaction between the professions, point to a special role of physicians in this context. This dominance was reasoned to be legally based. A physician elaborates on this within a group discussion:

*Back to the issue of who can call upon someone etc. if somebody has a different opinion on the therapy from a mere legal position the physician is held responsible, if it is about the kind of treatment or if you look at patients` complaints or the evaluation questionnaires, we take the stick and there are critics that are really below the belt and to more than 90 percent address physicians, not therapists or nurses. Or rather we as physicians have to take the can, maybe even with legal measures. That is why it is important that there is a certain hierarchy that is indisputable especially regarding the prescriptions.* (B3/GDST/HD/953–964)

Still, rehabilitation facilities with an upper and a lower level of patient success differ in how they deal with this exceptional position of the physicians. One example from a facility with a lower level of patient success has been presented above, where a nurse refrained from doing something potentially helpful for a patient with explicit reference to the fact that some actions or decisions can only be taken by physicians. In another example, a nurse pinpoints the exceptional role of physicians` prescriptions for the conduct of rehabilitation processes:

*Therefore, it is always the communication, that is the prescription, that is very important for us, yes, that we have a prescription by a physician for everything that we have to fulfil, yes, since, as stated, we have to work according to prescriptions, yes, we need the prescriptions for the patients.* (B1/GDST/FF/146–150)

This way of dealing was different in the rehabilitation facilities with an upper level of patient success. Here, we have found statements pointing to the necessity of mutual trust, which should form the basis for responsible decisions.

*Or trust among colleagues. That is what I think, is a much better expression than flat hierarchies. Trust among colleagues, or trusting interaction, professional trusting interaction, very important, yes.* (A1/GDST/HC/1211–1214)

Here, we observed discussions about the useful degree of delegation. Opportunities to voice onès position and to experience a defined space for self-determined decisions were more pronounced in these institutions. For example, a leading physician elaborates in an interview on the usefulness and necessity of delegating certain tasks to other professional groups:

*Well, for me it is currently a very important topic. We still have very traditional role understandings here, that are not sustainable any more and will not be possible in the future. Simply because we cannot manage it by the personnel (…). You can certainly find a number of tasks of physicians that can be delegated to well-trained, non-medical assistants. By the time we do have far-reaching concepts that claim nurses or others with a good medical education could do a sort of a pre-anamnesis. Certain documents to be sorted, asking targeted questions and, in a way, to prepare an extended patient record. And this could be the basis for the physicians consultation. It could within the logistics, the planning, regarding diagnostics and processes, a lot could be withdrawn from the physician, what is currently attached to the physician`s profession.* (A2/IVLP/536–555)

Interestingly, in the interviews with the leading physicians in the rehabilitation facilities with an upper level of patient success, we found statements on the need to improve interdisciplinary cooperation, which included a better integration of nursing staff in the rehabilitation team (a deficiency in all facilities we had visited).

*Well, the better the single professions are toothed or integrated, the better it is for the staff and the patients. Because the exchange of experiences can take place from any side, problems can be considered much faster, well, it would be also preferable if nurses could take part in our team meetings. Like that for example. We have done that before. But this is due to our low staffing. That is how one has to see that (…). If you could draw on unlimited resources, you could surely do even better than now.* (A3/IVLP/601–612)

This kind of statement regarding the *need* for improvement of interdisciplinary cooperation was not found in the rehabilitation facilities with a lower level of patient success.

### Different perspectives on hierarchies from the perspective of the staff members^3^

We found a different way of interaction between the participants of the group discussion (staff members) that could be related to different perspectives on hierarchies. In a separate step, we analyzed the group discussions of staff members more thoroughly (22). Staff members differ in how they personally and professionally relate to each other. On a personal level, positive experiences with colleagues were perceived as an expression of flat hierarchies. On a professional level, these hierarchical structures were interpreted differently. Especially in the rehabilitation facilities with a lower level of patient success, these hierarchical structures seemed to be taken for granted and accepted, respectively. In the rehabilitation facilities with an upper level of patient success, responsibilities assumed by physicians were perceived as embedded within a mutual professional exchange.

We could also observe differences in how the staff members participated in the group discussion. Interestingly, in the rehabilitation facilities with a lower level of patient success, aspects of professional hierarchies were addressed by persons at the upper level of the hierarchy, while those in the lower level of the hierarchy addressed mainly issues of personal relationships or topics related to work atmosphere. In the rehabilitation facilities with an upper level of patient success, no such division of content was present; group members of different hierarchy levels contributed in different ways to this discussion. Also, the description related to situations that were relevant to hierarchies differed. For example, while in a rehabilitation facility with an upper level of patient success, participants portrayed a situation how they negotiated a disputed topic; for participants in a rehabilitation facility with a lower level of patient success, it was of main importance to reach a consensus visible to others. In sum, communication in rehabilitation facilities with an upper level of patient success appeared to be less centered around physicians. Hierarchical structures appeared to be less pronounced and more directed toward one leading manager. Also, professional discussions about ways of treating patients seemed to be given more room for discussion.

## Discussion

In a systematic comparison of rehabilitation facilities with an upper and a lower level of patient success, we could depict a difference in the daily practices of interdisciplinary cooperation among team members. Physicians were found to play an important part in this game. Our point of departure was subtle differences in the representation of team members within team meetings, which were directly related to the perception of worth and respect toward one’s work. While the communication structure was more centered around the physicians in the rehabilitation facilities with a lower level of patient success, there was a higher level of mutual exchange on eyes’ level in the rehabilitation facilities with an upper level of patient success. This also related to perceptions of hierarchy in the rehabilitation team, which were more one-sided and physician-related in the rehabilitation facilities with a lower level of patient success. In these institutions, the physicians seemed to take a more dominant role in the team.

It should be noted that the only study that related organizational-level information to members of the rehabilitation team found a positive relationship between network social capital and patient outcome (21). Network capital represents the relational dimension of social capital in a working team. It can be understood as the feeling of cohesion, mutual support, and social fit. While we found this network of social capital also present in the rehabilitation facilities with a lower level of patient success, it seems to better fit a professional interaction model of mutual cooperation compared to a strictly hierarchical understanding with one-sided ways of interaction.

From a theoretical perspective, it seems appropriate to relate our results to the work of Strasser and Falconer (29) on team effectiveness in rehabilitation from the United States. As Strasser and Falconer (29), we saw a distinction between personal and professional relationships among team members. However, this distinction was more pronounced in those facilities with a lower level of patient success, while in facilities with a higher level of patient success, a more professional mutual respect model could be identified.

We found substantial convergence of our results with the team effectiveness model developed by Strasser and Falconer (see (30)). This model related team characteristics to patient outcomes. These characteristics are related to four dimensions: interprofessional relations, leadership, social climate, and managerial practices. Within interprofessional relations, we could show that mutual respect, trust, and the on-eyes-level professional relationship could be understood as a prerequisite for good patient outcomes. Leadership in our study meant the capability to foster this type of interprofessional relationship, to endure the tension to be aware of the leadership position, to take responsibility for final decisions, to take the blame, at the same time to allow for the different professions to integrate their competencies into the rehabilitation process, to provide mutual exchange, respect, and understanding, and to see clearly deficiencies of teamwork and opportunity to improve. With regard to social climate, it seems not to suffice that most team members feel well but to feel mutual trust, understanding, and eye-level respect. We did not focus on the issue of working on patient-centered goals, which is related to another result cluster of the project, but might acknowledge that this point might be of importance to the issue of social climate. With regard to managerial practice, we are not able to add to the theory at this point since the data-driven model presented by Smits et al. (30) was not realized in the facilities we have visited and does not seem to be common in German rehabilitation facilities—therefore opening up an important room for improvement related to patient outcome.

How could these results relate to success on a patient level? The team effectiveness model is not specific about how these characteristics identified on an organizational level actually translate to greater success on a patient outcome level. We have to delve much deeper into potential effective ingredients of rehabilitation care on the patient outcome level to answer this question. Presumably, we have only captured *potential prerequisites* of patient success, while further determinants and their interactions with team-level characteristics have to be identified on the patient level.

There are different limitations of our study. One point of critique may be related to our main reliance on patient-reported outcome data to appraise a rehabilitation facility as an upper or a lower level of patient success. While patient-reported outcomes are widely applied in clinical and health-related research as well as quality assurance, issues of validity have to be taken into account (31), especially concerning the purpose of the assessment (32). Also, other potential quality characteristics could have been used to identify and group the rehabilitation facilities, e.g., quality of peer-reviewed social medicine reports, possibly yielding different rankings of the clinics, and, therefore, possibly different results. However, by choosing an extreme group sampling design (i.e., upper and lower 10% of the distribution), we assume to have attenuated these concerns. This argument is based on the assumption that general quality aspects (related to very good or poor quality) should be expressed in very different indicators of quality. It could also be argued that outcome quality is the most important aspect of quality (the proof of the pudding), which guided the choice of our approach.

Also, we could argue that our study design follows a mere explorative approach, which is in line with the idea of ending up with empirically founded hypotheses as a study result. We can understand our study design as a case–control study comprising an *n* of 6—which does not look very convincing. However, we were able to dig into important mechanisms compatible with the literature on team effectiveness. It is highly plausible to assume that our results also apply to other rehabilitation facilities not included in our study or even to other patient groups cared for in rehabilitation facilities not considered in our study. It comes as no surprise that the results of our study were thoroughly discussed with a field of care not included in our study at all: patients with addictive behaviors and their respective rehabilitation care institutions (33). The empirical part of our study was conducted almost a decade ago. However, as we deal with very pervasive structural phenomena, we do not see a good argument to believe that the results are not as valid today as they were 10 years ago, as the pressures on the rehabilitation system to be as efficient as possible and to follow quite bureaucratic formal obligations continue to remain. In the meantime, professional training explicitly for rehabilitation teams has actually been established and evaluated (34) and is offered by the German Pension insurance.^4^ Also, recently interprofessional training with rehabilitation-related modules has been developed and evaluated (35).

## Data Availability

The datasets presented in this article are not readily available due to anonymity protection. Excerpts of the transcript are publicly available in Stamer et al. (18) and an additional accompanying report [see (18)]. Please contact the first author for further information. Requests to access the datasets should be directed to Thorsten Meyer, thorsten.meyer@uk-halle.de.

## Footnotes

^1^The acronym stands for characteristics (Merkmale) of a successful (erfolgreichen) rehabilitation facility (Reha-Einrichtung).

^2^For the unique identification of the citations and its sources we apply labels as follows: the first two signs represent the respective rehabilitation facility, “A” denotes to those with upper level of patient success, “B” to those with lower level of patient success, while the numbers 1–3 identify one of the three facilities in each group. “GD” denotes to group discussion, “IV” to an interview, “OP” to observational protocol; “ST” to staff, “PT” to patients, “LA” to leading administrator, and “LP” to leading physician. “F” then relates to a female, “M” to a male respondent, while the subsequent letter serves as an identity marker for a specific person. The last two numbers identify the line number of the respective transcript.

^3^An elaboration on the results of this topic, including transcripts of the interaction among the different professionals within the group discussions, have been published by Kleineke et al. (27).

^4^See, for example, www.deutsche-rentenversicherung.de/DRV/DE/Experten/Infos-fuer-Aerzte/Fort-Weiterbildung-Aerzte/fortbildung_sozialmedizin.html.
